# Surgery

**Published:** 1861-01

**Authors:** 


### Bi-Monthly Periscope.

### SURGERY.

SOME REMARKS ON A PECULIAR AFFECTION OF THE KNEE-JOINT. By *Francis James Lynch*, M.D., Physician to the Loughrea Workhouse and Fever Hospital.—Mr. Mayo, in the eleventh volume of the Medico-Chirurgical Transactions of London, directed the attention of the profession to a form of disease in the knee-joint, termed by him "An acute form of ulceration of the cartilages of the joints;" and, subsequently, in his work on Human Pathology, p. 90, he further alludes to it, under the heading of "A class of cases of rare occurrence." Sir Benjamin Brodie, Mr. Hawkins, Mr. Key, Mr. Wickham, and others, have since published remarks on the subject; and most surgeons in Great Britain and Ireland in extensive practice are, doubtless, familiar with the disease, and its appropriate treatment; still, as it is rather a rare form of disease, the following observations and cases may not be wholly devoid of interest.

The disease is mostly observed in persons who have been subject to rheumatism, or to rheumatic gout, and is often brought on by exposure to damp and cold; the patient, after suffering from more or less acute synovial rheumatism of a migratory character, in the wrist, elbows, knees, and other joints, will suddenly complain of intense pain in one knee, where the inflammatory action seems to concentrate itself. The limb is almost always in the extended posture, the heel resting on the bed. The swelling, even at the commencement, is considerable, occupying the knee, the lower third of the thigh, and the upper third of the leg; it is not circumscribed above or below, but gradually tapers away, and is lost in the thigh and leg. The skin covering the affected part of the limb has a remarkably glossy, bloodless hue, resembling in color white marble. It is exquisitely tender to the touch, and the swelling is found to be uniform, firm, and elastic; sometimes a crackling is felt under the examining finger, and at times slight œdema, or pitting on pressure, exists. The joint is the seat of constant pain; sometimes dull and throbbing, at other times lancinating and severe, with frequent and intensely painful exacerbations. The tenderness is very great, even in the skin, and the least pressure cannot be borne. Moving the toes, or shaking the bed, or walking heavily across the room—in fact, whatever occasions the slightest disturbance or movement of the limb—causes intense agony. There does not seem to be much, if any, effusion into the joint; and the usual prominences about it cannot be easily detected, owing to the thickened condition of the soft parts, and the extreme sensitiveness of the skin. Frequent spasmodic twitches in the limb increase the patient's sufferings, and interrupt the snatches of sleep, which anodynes, or exhausted nature, at times induce. From the commencement, there is a good deal of febrile excitement; nausea, anorexia, thirst, disturbed rest, quick pulse, heat of skin, and other marks of constitutional derangement, exist; the countenance is expressive of much pain and anxiety, and, as the disease progresses, emaciation, increasing debility, perspirations, and frequently recurring rigors, are observed. In this condition things may remain for weeks or months, when, in the majority of cases, either from the effects of remedies, or, in rarer cases, from apparently the unaided efforts of nature, the urgent local and constitutional symptoms gradually or abruptly abate, and the patient ultimately recovers, with the joint apparently anchylosed; but in process of time a limited degree of motion sometimes returns, only to a slight amount. Ultimately, however, after a year or two, the patient is able to walk without much lameness. The swelling never completely subsides; the patella is more or less fixed; the hollows on either side of the ligamentum patellæ are permanently filled up, not from effusion into the joint, but from thickening of the exterior tissues.

Owing to the limb resting for such a length of time in the extended posture, troublesome sores and excoriations are apt to form on the heel and nates; for months after the subsidence of the acute symptoms the patient is unable to rest on the limb, and its premature use will surely reproduce the original symptoms. Occasionally, during the acute stages of the disease, a degree of tenderness and some fullness is felt in the course of the femoral vessels as high as the groin, where an enlarged gland can be sometimes felt; showing that, in some instances, the venous and absorbent vessels participate in the existing inflammatory action. Such is the progress and termination of the disease, in its milder and most favorable forms; its leading features being well summed up by Mr. Hawkins in the following extracts from one of his clinical lectures on the subject, viz.: "1. Very acute tenderness of the skin, resembling, in this respect, hysterical affections of the knee. 2. Great aggravation of pain on moving the limbs, or the bed on which they lie. 3. A pale, white, glossy appearance of the skin. 4. The swelling is peculiar, differing from that of simple synovial inflammation; for, while the swelling in the latter extends around the joint alone, in this disease it extends around the lower part of the thigh, and the upper part of the leg; it pits on pressure; and you can feel a sensation of crackling beneath the finger." Mr. Hawkins believes that the disease commences in the periosteum, covering the lower end of the femur; and that after involving the areolar membrane external to the capsule, it passes thence to the synovial membrane and cartilages of the joint. At all events, it is probable that the inflammation originates in the areolar and fibrous tissues external to the joint. If, as occasionally happens, the disease is supposed to be rheumatic gout, or common synovial rheumatism, and is treated by colchicum, and afterward by hydriodate of potash, the patient will suffer for months from the violence of the pain, and the attending irritative fever, escaping ultimately with a joint more or less completely anchylosed, or the cartilages and synovial membrane of the joint become ulcerated and disorganized; and matter forms in and around the joint, running down the patient's strength, and either ending fatally, or rendering amputation of the limb necessary for the preservation of life. In the vast majority of cases, however, there is no tendency to the formation of matter within or around the joint; the cartilages are absorbed; and false anchylosis, without any accompanying suppuration, is established. If the disease be early recognized, rapid mercurialization of the system, and local depletion, will quickly restore the joint to its normal condition; even at a more advanced period, the same line of treatment will, in the vast majority of cases, arrest further mischief, and relieve in a very marked and satisfactory manner the local and constitutional symptoms, which begin abruptly to disappear as soon as the mercurial action is established. The painful condition of the limb pending the mercurialization of the system is often remarkably relieved by local sedative applications, among the most potent of which is a lotion of cyanide of potassium dissolved in water, (four grains to the ounce,) applied tepid. In general, warm applications are preferred to cold; and French wadding, or carded wool, around the joint and limb, with a pad under the heel, will be grateful and useful. Generally speaking, counter-irritation, by blisters or otherwise, does more harm than good, even on the decline of the disease. When the acute symptoms have wholly subsided, and the patient is able to rest a little on the limb, the Buxton waters and a judicious course of shampooing tend powerfully to restore a certain degree of motion to the joint. During convalescence, there are sometimes returns of severe pain, which are best relieved by the cyanide of potassium lotion, or by belladonna—two drachms of the extract in an ounce of water, smeared over the seat of pain.

In the acute stage, the stomach is so much disordered that opium, in any shape, does more harm than good, when taken internally to relieve pain or produce sleep; but I have found Battley's sedative liquor to be, when a sedative is urgently required, the most preferable form, far more so than any of the preparations of morphia. During convalescence, profuse perspirations are common; here the liquor cinchonæ, and eight grains of tannin, every night, will often check this exhausting symptom. A flannel bandage, using very moderate pressure, from the toes upward, is useful; but bandaging should not be attempted until *all* pain and tenderness cease. When the disease rapidly disorganizes the cartilages and other textures of the joint, or when a similar result follows a protracted attack, the case assumes the features of ordinary acute or sub-acute ulceration of the cartilages of the joint, and requires a similar treatment.

There is a well-known form of acute inflammation, which has been well described in this journal by the late Mr. M'Dowel, attacking the periosteum and articulating ends of the bones—in the vicinity of some of the larger joints, in certain states of the system, rapidly extending to the synovial membranes and cartilages of the contiguous articulations, and ending speedily in purulent effusion into and around the joint, absorption of the cartilages, necrosis of and separation of the epiphyses of the bones, thickening and separation of the periosteum, and other morbid changes, which is not to be confounded with the disease under consideration; such cases occur after parturition, or as a sequel to scarlatina, erysipelas, small-pox, typhus fever, or what has been termed diffuse inflammation. Their course is rapid: several articulations are attacked in succession; a blush of redness, sometimes faint, sometimes vivid, is observed on the surface: the febrile disturbance quickly assumes the typhoid rather than the irritative character; and mercury and all other modes of treatment are mostly useless.

The following cases, illustrative of the above remarks, are extracted from various papers on the subject, and a few of them have occurred in my own practice. The disease may, doubtless, affect other joints, but I have only seen it in the knee; and it cannot be very common, as, in twenty-five years' extensive experience, I have only met with three or four instances of it.

A married female, aged forty, after suffering from an attack apparently of rheumatic gout, flying about from one joint to another for several weeks, was suddenly seized with violent pain in the right knee-joint, accompanied by swelling and great tenderness. Her medical attendant in vain pressed the mercurial treatment, it would not be listened to, and the lady continued to suffer severely for some weeks. Colchicum, hydriodate of potash, and local depletion having been had recourse to without any advantage, I found her, about the third week of the disease, in the following condition: The knee was much swollen, the swelling being greatest at the joint, and tapering upward as far as the middle third of the thigh, and downward to the calf of the leg. The integuments are of a pale, glossy, white color, without a trace of redness; exquisitely tender to the touch, particularly over the joint; and rather hot. There is a severe throbbing pain in the joint, chiefly felt at its inside, and much increased by the slightest motion of the limb, or by walking heavily across the floor; occasionally, violent pain of a lancinating character darts across the joint. The swelling is elastic, free from any enlarged veins, and does not pit on pressure. A good deal of tenderness exists along the femoral vessels, to the groin, where a few enlarged and tender glands can be felt. The natural projections and depressions around the joint are obliterated, but apparently more from effusion outside than within the joint. The limb lies in the extended posture, and the patient dreads the slightest movement of it, or of the adjoining portion of the bed. The heel is very painful, owing to the motionless condition of the limb. There are frequent spasmodic twitches in the muscles; and the entire limb feels hot, but seems cold to the patient, so as to oblige her to keep it wrapped up in flannel and carded wool. There is great uneasiness in the popliteal region—a feeling of distention there, as if the skin would burst. There was total want of appetite; great thirst; evening exacerbations, characterized by heat of hands, flushing of face, and increased heat of skin. She had chills every day, and perspired copiously at night; the pulse was 100; she had got very thin; slept very badly; and often suffered from nausea and vomiting. Pressure along the surface of the tibia, and over the femur, where it is most superficial, caused great suffering. The countenance was expressive of much anxiety; and bedsores were beginning to form.

Fomentations and leeches gave temporary relief; opium, in any shape, could not be borne; three grains of blue pill were given every four hours; the bowels were attended to; and a lotion composed of sixteen grains of cyanide of potassium and four ounces of distilled water applied tepid, whenever the pain becomes severe.

During the next five or six days, there was but little change in the patient's condition; but extraordinary relief to the pain followed each application of the lotion—she called it the magical bottle.

On the seventh day moderate ptyalism set in, and was followed by complete relief to the pain, and speedy subsidence of the constitutional disturbance.

A long period, however, elapsed before the patient could leave her bed. Blisters, to accelerate convalescence, did no good; and a very cautious application of pressure and support, by means of suitable padded splints and bandages, after the acute symptoms had long completely disappeared, could not be endured. Later still, a journey to Buxton was with difficulty accomplished. Considerable advantage was derived from the use of the waters; but more particularly from a well-directed course of shampooing.

Three years, however, after the first onset of the attack, and notwithstanding the most careful attention, the motions of the joint were very imperfectly executed; and, although the lady walked without a stick or support, the knee could be only very slightly flexed, and the patella was only very partially movable.

Jane Williams was admitted into the Loughrea Union workhouse, on the 10th of June, 1856. On admission, the right knee was very much swollen, exquisitely tender to the touch, of a pale glossy hue; the swelling being greatest at the joint, and extending upward and downward for some inches, gradually diminishing, until it was ultimately lost in the thigh and upper third of the leg. The skin over the seat of the swelling was perfectly colorless, and presented an appearance very similar to what is observed in phlegmasia dolens. There was no œdema, the swollen parts being rather springy and elastic. The shape of the joint was completely lost, the usual depressions and prominences being obliterated. The patient lay on her back, with the limb in the extended posture, and could not endure the slightest change of position either of the limb or body; there were occasional sharp pains darting through the limb and joint; and a dull constant feeling of uneasiness, more than pain, was felt in it. The sleep was much disturbed by painful startings of the limb; toward morning, profuse perspirations occurred, and at several periods during the day alternate chills and flushes of heat were perceptible. The pulse was 120, and weak; the countenance anxious and depressed; the appetite wretched; much emaciation and debility existed; there were thirst, despondency, occasional nausea, and rejection of food. The patient was a strong, middle-aged woman, who had several attacks of migratory, but not severe rheumatism. A month before admission she caught cold, and was seized with violent pain, accompanied by swelling of several joints in succession. After struggling for a month against the disease, without medical assistance, the pains left the other joints, and the right knee became suddenly swollen, and the seat of acute pain.

Two grains of calomel, with one-fourth of a grain of opium, given three times a day, produced ptyalism in six days, when all painful feelings in the joint and limb completely and suddenly ceased; but a considerable degree of tenderness existed for months, and several weeks elapsed before the limb could be put to the ground. After some months, strapping the limb with plaster of gum-ammoniac with mercury, spread on leather, seemed to give much relief and support to the limb, coupled with a well-adjusted bandage. A moxa, and afterward a caustic issue, were tried without advantage; but ultimately she acquired considerable use of the limb, so as to be able to walk without much lameness, and without support. But after an interval of four years, when I again saw the patient, the joint was imperfectly anchylosed, little motion existed, and the patella was only partially movable.

As I have already mentioned, the disease generally terminates, in its milder forms, by a gradual disappearance of all the symptoms; more frequently, anchylosis, to a greater or less extent, results; in other instances ulceration of the cartilages of the joint, without any formation of purulent matter, ensues, giving rise to febrile symptoms, first of an inflammatory, then of a typhoid character, which speedily run down the patient, and destroy life, even in cases where active and appropriate treatment has been employed from the onset. The following case illustrates this latter mode of termination.

A girl, aged twenty-two, liable to rheumatism, and accustomed to vicissitudes of temperature, admitted under Sir B. Brodie, into St. George's Hospital, in June, 1827, after laboring under rheumatic pain in the elbows and shoulders for a month, was suddenly seized with exquisite pain in the right knee, increased on pressure, and aggravated by every motion of the joint. The pain was principally referred, not to the knee itself, but to the thigh-bone immediately above it. The swelling extended from the upper part of the thigh to the leg below the knee, being most conspicuous in the immediate neighborhood of the knee-joint, and thence gradually diminishing, above and below, without any defined termination. The swelling was very considerable, glossy, tense, elastic, under-colored, exquisitely tender to the touch, resembling in color white marble; and both the pain and enormous swelling had occurred suddenly, the day before. There were quick pulse, hot skin, brown furred tongue, and a countenance expressive of anxiety and suffering.

Three weeks' treatment by leeches, salines, calomel, and opium nearly removed all the symptoms, both local and constitutional. Accidental exposure to cold, however, reproduced them; and, notwithstanding a repetition of the treatment which had been before successful, along with blisters, and the establishment of an issue, painful startings of the limb ensued, and other symptoms of the ulceration of the cartilages, which were rapidly undermining her strength, when an attack of peritonitis carried her off, in two months from the commencement of her illness.

On dissection, the cartilages investing the condyles of the femur, the head of the tibia, and the patella, were ulcerated, and the cancelli of the bones exposed beneath; the periosteum was easily separable from the bone for some distance up, and the bone itself was vascular. In this case it is probable that recovery would have taken place, without much, if any injury to the point, had not a relapse from imprudent exposure to cold taken place, as the treatment was prompt and apparently successful at first. I should mention, that not a particle of purulent matter was found within or around the joint, but some blood was effused into it. Although, in general, there is not much tendency to the formation of purulent matter in the milder forms of this disease, many cases are recorded of its running a much more rapid and destructive course, disorganizing the joint, and occasioning extensive purulent depositions in the joint and limb, either among the muscles, or between the periosteum and bone, or in all three situations. Thus, in a case adverted to by Mr. Hawkins in the twelfth volume of the *Medical Gazette*, and in which the symptoms were exactly similar to the last case, as regards the local symptoms and constitutional disturbance, the patient gradually sank, after much suffering; and, on dissection, the cartilages were found to be extensively ulcerated; there was no purulent matter within the joint, although a good deal of matter was found, nearly the whole length of the thigh, among the muscles, near the bone. The periosteum of the femur was much thickened and condensed for some distance above the knee, and the bone was vascular. In another instance of this severe form of the disease, narrated in the 24th volume of *Johnson's Medico-Chirurgical Review*, p. 23, the patient died in a typhoid condition; and, on dissection, the principal seat of the disease was found to be in the periosteum and areolar membrane immediately exterior to it; the former was stripped from the femur throughout its lower half, and was lying loosened and dead in the midst of pus; the articular cartilages were ulcerated; the joint contained pus, which communicated, by an ulcerated opening through the synovial capsule, with matter on the exterior of the joint. In the following case, extracted from Mayo's work on Pathological Anatomy, the disease ended favorably in anchylosis, apparently uninfluenced by treatment: A young woman, twenty-seven years of age, six months after labor, was seized with pain and swelling of the wrist; the next day, the right knee was attacked in like manner. The following day, the pain and swelling left the joint first affected, and attacked and settled in the left knee. Six weeks after the commencement of her illness, Mr. Mayo saw the patient. The thigh and leg were swollen, with serum effused into the areolar membrane; the joint contained no fluid; the pain in the knee was very acute and constant, and increased by the least motion; the patient lay on her back, with the knee straightened; leeching gave no relief; cold applications and fomentations were equally inefficacious; repeated blistering appeared at one time to be of service; small doses of calomel and opium were administered frequently during a few successive days, with no advantage; at length the pain seemed to abate spontaneously; the swollen state of the limb gradually subsided; the patient recovered the appearance of health, but the joint was wholly immovable.

Mr. Syme, in the number of the *Lancet* for April 19th, 1856, p. 437, makes some remarks on a form of articular disease which has some points of resemblance with the affection I have attempted to illustrate. Professor Syme's description is as follows: The disease is generally met with in rheumatic subjects, and, after shifting from one place to another, fixes itself in the affected joint. In other cases it appears suddenly, and as a sequel of some feverish disturbance; it is hardly ever met with in the ball-and-socket joints, preferring the hinge-joint articulations.

The symptoms are: severe, deep seated pain, aggravated by pressure or motion, with exacerbations at night, and on the approach of rainy weather; diminution or complete loss of muscular power, with uneasiness in the limb generally, or in the nearest joint; numbness, or want of proper sensation; more or less swelling, of an unyielding kind, confined to the seat of the articulation. It ends, after weeks or months, in recovery, with more or less stiffness in the joint, or in suppuration and ulceration of the cartilages. When the joint is examined at an early period, the cartilages are found eroded, with a little bloody fluid in the cavity of the joint. Internal medicines, the usual local remedies, such as fomentations, poultices, leeches, blisters, and issues, are useless; but the application of the actual cautery, used while the patient is under the influence of chloroform, is said to be wonderfully efficacious. The disease which Mr. Syme describes seems to be confined to the synovial membrane and cartilages of the joint, and to be unaccompanied by any diseased condition of the periosteum, or of the bone, in the immediate vicinity of the articulation.—(*Dublin Quarterly Journal of Medical Science*, November, 1860.)

